# Determinants of per diem Hospital Costs in Mental Health

**DOI:** 10.1371/journal.pone.0152669

**Published:** 2016-03-31

**Authors:** Jan Wolff, Paul McCrone, Anita Patel, Claus Normann

**Affiliations:** 1 King’s College London, Institute of Psychiatry, Psychology & Neuroscience, King’s Health Economics, De Crespigny Park, London, United Kingdom; 2 Medical Centre-University of Freiburg, Department for Management and Controlling, Freiburg, Germany; 3 Queen Mary University of London, Barts and the London School of Medicine and Dentistry, Centre for Primary Care and Public Health, London, United Kingdom; 4 Medical Centre- University of Freiburg, Department of Psychiatry and Psychotherapy, Freiburg, Germany; Deakin University, AUSTRALIA

## Abstract

**Introduction:**

An understanding of differences in hospital costs between patient groups is relevant for the efficient organisation of inpatient care. The main aim of this study was to confirm the hypothesis that eight a priori identified cost drivers influence per diem hospital costs. A second aim was to explore further variables that might influence hospital costs.

**Methods:**

The study included 667 inpatient episodes consecutively discharged in 2014 at the psychiatric hospital of the Medical Centre- University of Freiburg. Fifty-one patient characteristics were analysed. Per diem costs were calculated from the hospital perspective based on a detailed documentation of resource use. Mixed-effects maximum likelihood regression and an ensemble of conditional inference trees were used to analyse data.

**Results:**

The study confirmed the a priori hypothesis that not being of middle age (33–64 years), danger to self, involuntary admission, problems in the activities of daily living, the presence of delusional symptoms, the presence of affective symptoms, short length of stay and the discharging ward affect per diem hospital costs. A patient classification system for prospective per diem payment was suggested with the highest per diem hospital costs in episodes having both delusional symptoms and involuntary admissions and the lowest hospital costs in episodes having neither delusional symptoms nor somatic comorbidities.

**Conclusion:**

Although reliable cost drivers were identified, idiosyncrasies of mental health care complicated the identification of clear and consistent differences in hospital costs between patient groups. Further research could greatly inform current discussions about inpatient mental health reimbursement, in particular with multicentre studies that might find algorithms to split patients in more resource-homogeneous groups.

## Introduction

Understanding the differences in hospital costs between patient groups is relevant at different levels of health care systems. This can be decision-making in clinical practice or the efficient allocation of funds at policy level, for instance by the design of reimbursement schemes.

A well-designed reimbursement scheme is necessary for the efficient delivery of inpatient care. Inappropriate reimbursement rates can create unintended incentives, such as the reduction of costs at the expense of quality, early discharges to reduce length of stay for pecuniary reasons or to avoid treating high cost patients [1–3]. There are at least two reasons why such incentives can be stronger in mental health care than in medical and surgical care [4]. First, there is a wider range of accepted treatment practices enabling providers to choose based on financial incentives. Second, psychiatric hospital care can be prone to undertreatment since its quality is more difficult to measure.

Case-mix based reimbursement has gradually become the basis of paying hospitals in most industrialised countries [5]. Such schemes prospectively define hospital payment rates specifically for patient groups of assumed homogeneous resource use [6]. Most case-mix based reimbursement schemes in medical and surgical care set hospital payment rates for a complete inpatient case [7]. However, it has been very difficult to infer length of stay from patient characteristics in mental health care, which makes defining groups of homogeneous resource use *per case* infeasible [8]. Therefore, attention has shifted more towards prospective payment systems that provide per diem payments and several large scale studies were carried out that analysed the feasibility of such systems [9–11]. Furthermore, prospective per diem payment systems are used in the USA and are currently being implemented in Germany. These systems employ per diem rates that decline with increasing length of stay, which create an incentive to reduce length of stay, but less strict than created by per case payments.

Prospectively defining adequate per diem payments for different patient groups requires an understanding of factors that influence per diem hospital costs. A systematic review has found six patient and two service characteristics that influence per diem hospital costs [12]. However, most included studies were old and only one small study was carried out in a European hospital setting. A recent primary study carried out in a European hospital setting has found substantial differences in per diem staff costs between wards for different patients groups [13]. However, it was restricted to staff costs and did not analyse the individual influence of patient characteristics.

The aim of this study was to confirm the hypothesis that the eight concepts a priori identified as potential cost drivers in a systematic review [12] influence mean per diem hospital costs per inpatient stay in a current European hospital setting. These were 1. age, 2. major diagnostic group, 3. risk, 4. legal problems, 5. the ability to perform activities of daily living, 6. the presence of psychotic or affective symptoms, 7. the day of stay and 8. the treatment site. A second aim was exploring further patient variables that might influence mean per diem hospital costs.

## Methods

The study included all inpatient episodes (n = 667) discharged in 2014 at the psychiatric hospital of the Medical Centre- University of Freiburg, Germany. An episode was defined as the time from admission to formal discharge. Readmission after formal discharge and the initial episode were treated as an aggregated episode if time away was less than two weeks. Episodes treated at the ward for privately insured patients were excluded because it was not possible to collect all data for these patients. The hospital has eight wards with a total of 120 beds. The study and its methodology were approved by the works council of the Medical Centre-University of Freiburg. The ethics committee of the Albert-Ludwigs-University of Freiburg confirmed that the study did not require formal approval. All data were analysed anonymously.

### Data

The analyses required both a description of episode-specific patient and service characteristics and episode-specific mean per diem costs. Patient characteristics were derived from the electronic medical records of the psychiatric hospital and from the patient administration database. A total of 51 patient characteristics were investigated. These included the variables from the admission module of the minimum data set recommended by the German Association for Psychiatry, Psychotherapy and Psychosomatics [14]. Furthermore, a nursing assessment of characteristics relevant for the care process and further medical and demographic data relevant for the administration were available. A full list of all investigated patient characteristics can be found in Table 1 and S1 Table in the electronic supplement to this article.

**Table 1:** Basic patient and service characteristics.

number of patients		593
number of episodes		667
length of stay, median in days (iqr)		51 (52)
age, mean in years (sd)		45 (17)
female, percentage		59
Independent variables used in mixed-effects model	n	%
Age, years		
<33 or >64	371	66
33–64	296	44
missing	0	0
ICD-10, main diagnosis		
F1 Substance	84	13
F2 Psychotic	86	13
F3 Affective	339	51
F4 Neurotic	64	10
F6 Personality	35	5
Others	59	9
missing	0	0
Danger to self		
no	517	78
yes	136	20
missing	14	2
Danger to others		
no	647	97
yes	14	2
missing	6	1
Involuntarily admitted		
no	612	92
yes	48	7
missing	7	1
ADL, eating		
no problems	317	48
problems	331	50
missing	19	3
ADL, toileting		
no problems	571	86
problems	77	12
missing	19	3
ADL, mobility		
no problems	477	72
problems	171	26
missing	19	3
ADL, hygiene		
no problems	497	75
problems	151	23
missing	19	3
Delusional symptoms, AMDP-Score		
not existent (0)	531	80
light (1)	58	9
medium (2)	43	6
severe (3)	29	4
missing	6	1
Affective symptoms, AMDP-Score		
not existent (0)	21	3
light (1)	136	20
medium (2)	183	27
severe (3)	321	48
missing	6	1
Length of stay		
1–35 days	436	65
>35 days	231	35
missing	0	0
median of total costs per episode in € (iqr)		11,764 (12,180)
median of mean per diem costs in € (iqr)		240 (79)

sd = standard deviation, iqr = interquartile range, ICD = International Classification of Diseases, AMDP = Association for Methodology and Documentation in Psychiatry.

The eight concepts that were identified as potential cost drivers in advance were operationalised in this paper with the aim to be as close as possible to the results of the systematic review [12] with the available data. Therefore, *age* was dichotomised and patients of middle age, meaning 33–64 years, were separated from other patients, since the systematic review identified middle-aged patients to be different from young and old patients. *Risk* was operationalised with two variables, i.e. danger to self and danger to others, separately. Being admitted involuntarily or by a legal guardian was used to represent the concept of *legal problems*. Any problems in the dimensions eating, toileting, mobility and hygiene were separately used to represent the *performance in activities of daily living*. The score for delusional symptoms and the score for affective symptoms on the scale of the Association for Methodology and Documentation in Psychiatry (AMDP) were used to represent the *degree of psychotic or affective symptoms* independently from each other and from the actual main diagnosis. Furthermore, the aim of this study was to estimate mean per diem hospital costs. Therefore, the concept *day of stay*, i.e. cost at day one versus cost at day two, etc., was operationalised by the total length of stay of a hospital episode. Moreover, since this study was carried out at a single hospital, the concept *treatment site* was operationalised by the different wards at the hospital. These wards were led by different head psychiatrists and head nursing staff and were non-exclusively dedicated to different patients groups, such as patients requiring a locked environment, older patients and patients of specific diagnoses.

Per diem costs were calculated from the hospital perspective. All services a patient received during a stay were valued by their full operating costs to the hospital, i.e. including variable and fixed cost shares. This included all staff, materials, infrastructure and overhead costs at the psychiatric hospital as well as all costs for services from other departments, such as radiology and laboratories. Capital costs were excluded. The sum of costs an inpatient episode incurred was divided by the length of stay to calculate mean per diem costs.

Staff time in therapeutic interventions was calculated by multiplying the duration of each intervention with the number of therapists and dividing the product by the number of patients. This included psychotherapeutic interventions, physical therapies, occupational therapies and other measures, such as electroconvulsive therapies.

Staff time for regular care, meaning all clinical time except of therapeutic interventions, was calculated in a two-stage approach. First, total staff time in regular care activities was calculated for each ward in a two-week work sampling study including all physicians, nurses and psychologists involved in clinical care during the study period. The applied methods were described in detail elsewhere [13]. Second, total staff time in regular care was proportionally allocated among episodes using a daily documented three-dimensional acuity score, representing the 1. psychiatric, 2. somatic and 3. social effort of regular care, with three levels on each dimension [15]. Staff time in therapeutic interventions and regular care was monetarily valued, including non-wage labour costs, according to the salary and working hours defined for the year 2014 by the collective bargaining agreements applicable to the university hospitals in Baden-Wuerttemberg, Germany.

The consumption of expensive pharmaceuticals of at least 10 € per daily dosage was extracted from the electronic medical records and valued by their average purchasing price to the hospital’s pharmacy in 2014. Per diem costs for low price pharmaceuticals and other medical products were calculated by dividing the annual sum of such costs at each ward by the total number of patient days at each ward.

Ward-specific mean per diem overhead costs, such as buildings, beds, meals and administration, were derived by the iterative step-down allocation method on a cause-and effect basis with per diem charges. Services from other departments, such as radiology and laboratories, were documented by their local clinical sub-systems and valued by department-specific costs.

### Analysis

Fourteen per cent of total episodes had incomplete data, i.e. at least one variable missing. Multiple imputations by chained equations were used for the non-monotone pattern of missing data. The imputation model comprised all available independent variables and the outcome variable to prepare the data set for the explorative analysis. Twenty imputations with 50 iterations each were carried out, convergence of iterations was traced and distributions of imputed and observed data were compared.

The hypothesis driven analysis of variables previously identified as potentially relevant cost drivers was carried out using mixed-effects maximum likelihood regression. A three-level model was employed, with random intercepts for the individual episode nested within the patient and the patient nested within the discharge ward. The association of length of stay and mean per diem costs was assessed using partial residual plots and locally weighted regressions. The independent variables that were used to represent the eight potential cost drivers were checked in advance for potential multicollinearity with unproblematic results according to common standards [16] (mean VIF = 1.56, Condition number = 13.31).

The importance of all 51 available patient characteristics as potential cost drivers was explored using ensembles of conditional inference trees based on single stochastic imputation [17]. Conditional variable importance was calculated to avoid bias related to the previously identified multicollinearity in the 51 variables (mean VIF = 1.67, Condition Number = 46.43) [18]. The conditional variable importance is a measure that represents the relative importance of predictor variables included in ensembles of conditional inference trees. The importance measure represents the degree to which the predictor variables are associated to an increase in the predictive power of the inference trees. Therefore, it can represent both increases and decreases in per diem costs associated with the predictor variable.

Furthermore, a single conditional inference tree was estimated to identify potential patient groups for reimbursement purpose. Conditional inference trees recursively partition the sample into smaller subgroups by identifying the independent variables that best classify episodes into subgroups of ‘homogeneous’ per diem costs. At each split, the variable with the strongest association with per diem costs was selected based on the smallest p-value derived from permutation tests. The recursion was stopped when the algorithm failed to reject the Bonferroni-adjusted global null hypothesis of no significant association between per diem costs and any of the 51 patient characteristics at the 95% confidence level.

## Results

The study included 667 inpatient episodes with a median length of stay of 51 days. Table 1 shows basic patient and service characteristics. The largest diagnostic group was affective disorders, accounting for 51% of total episodes. The median total costs per episode were 11,764 €. The median of episodes' mean per diem costs was 240 €. Furthermore, Table 1 shows the independent variables used to represent the eight a priori identified cost drivers and the frequency of their values observed in the sample. The frequency of values in patient characteristics that were investigated later in the explorative analysis and that are not listed in Table 1 can be found in S1 Table.

**Fig 1:**
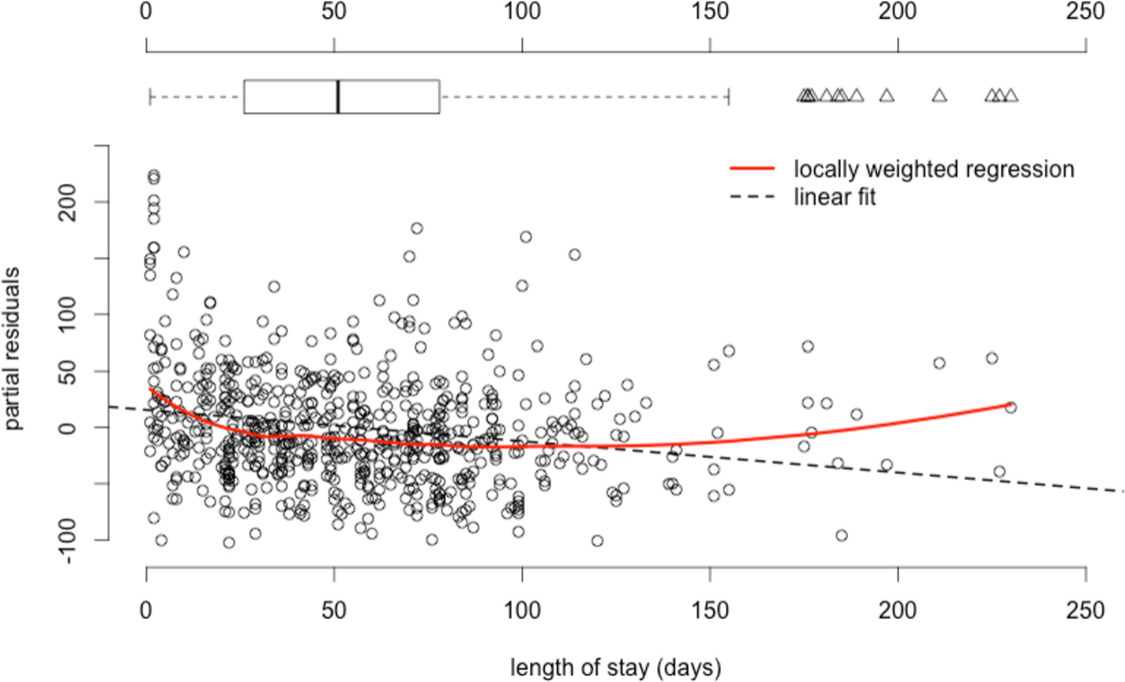
Partial residuals of mean per diem costs versus length of stay.

Fig 1 shows the association between observed lengths of stay and mean per diem costs in a component-plus-residuals plot. It plots the observed length of stay against the residuals of regressing per diem costs on the variables that represented the eight potential cost drivers plus the component associated with length of stay. Controlling for the other variables, per diem costs declined until a total length of stay of about five weeks. Thereafter, they remained mainly constant. An increase in per diem costs in episodes with a length of stay above 150 days was associated with a few outlier episodes, as indicated by the superimposed box-plot. Therefore, length of stay was dichotomised in the following analyses and patients staying no more than five weeks were separated from others.

**Fig 2:**
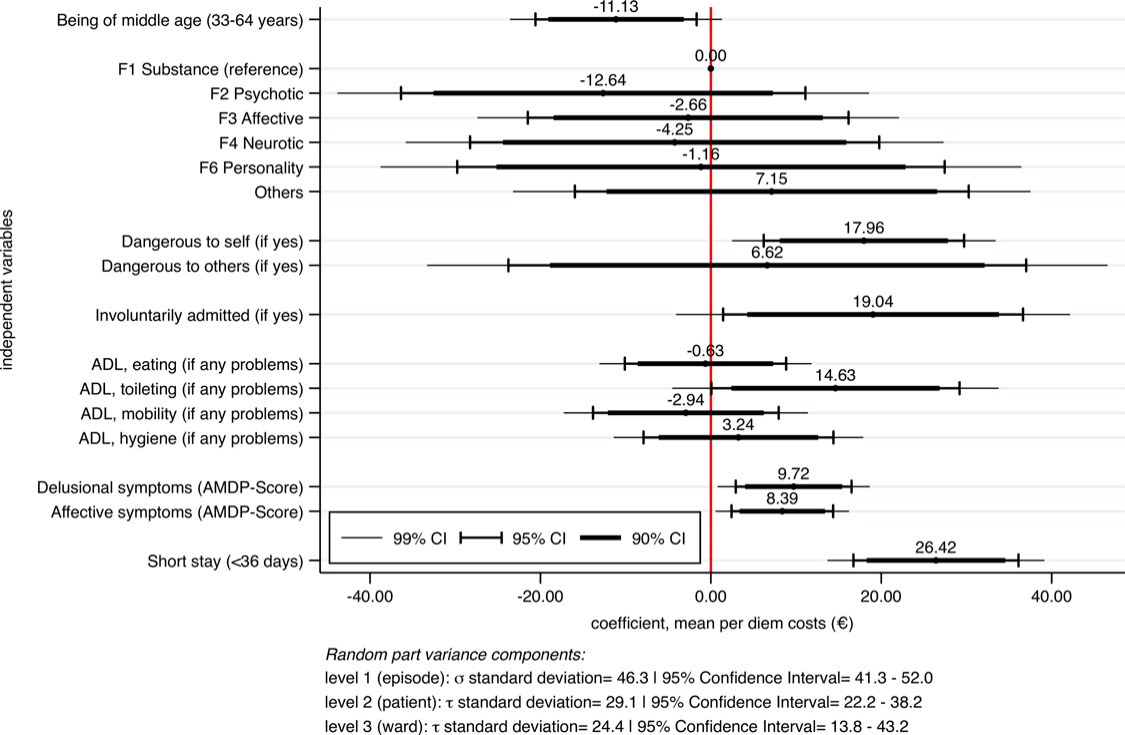
Fixed coefficients in mixed-effects maximum likelihood regression of mean per diem costs. F-groups represent main diagnosis and were derived from the International Classification of Diseases German Modification (ICD-10-GM), ADL = Activities of Daily Living, AMDP = Association for Methodology and Documentation in Psychiatry, CI = Confidence Interval.

Fig 2 shows the fixed coefficients in a mixed-effects maximum likelihood regression of per diem costs on the variables that represented the a priori identified potential cost drivers. The discharge ward represented the treatment site and was included in the model as random intercept. The variance components of the random effects are provided below the plot. Significant increasing effects on per diem costs were found in experiencing a short stay, followed by involuntary admissions, dangerousness to self, any problems in toilet hygiene and the degree of delusional and affective symptoms. Significant decreasing effects were found in being of middle age, i.e. between 33 and 64 years. Furthermore, the discharge ward showed significant and substantial variance in per diem costs.

**Fig 3:**
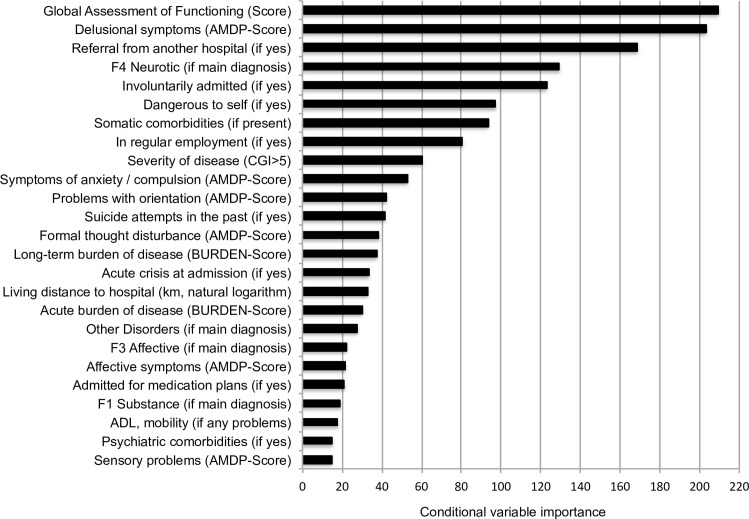
Conditional variable importance of explored patient characteristics. AMDP = Association for Methodology and Documentation in Psychiatry, CGI = Clinical Global Impression, km = kilometres, ADL = Activities of Daily Living, F-groups were derived from the International Classification of Diseases German modification (ICD-10-GM).

Fig 3 shows the relative conditional variable importance of the 25 most important variables in the prediction of per diem costs derived from an ensemble of 2,000 conditional inference trees. A figure of all 51 patient characteristics can be found in the supplementary material in S1 Fig. The absolute values of importance are neither informative by themselves nor comparable to other studies. Instead they should be used to compare the relative importance between variables under investigation [19]. The most important variable was the Global Assessment of Functioning (GAF) [20], followed by delusional symptoms, being referred from another hospital and neurotic disorders as main diagnosis. A rather steep decline in importance was found in the top ten variables. Thereafter, there were smaller differences in importance between variables. A prediction based on all 51 available variables yielded an explained variance of 19.4% and a Root-Mean-Squared-Error (RMSE) of 59.5 in out-of-bag internally cross-validated predictions [21]. Without cross-validation, an explained variance of 43.5% and a RMSE of 49.8 was found.

**Fig 4:**
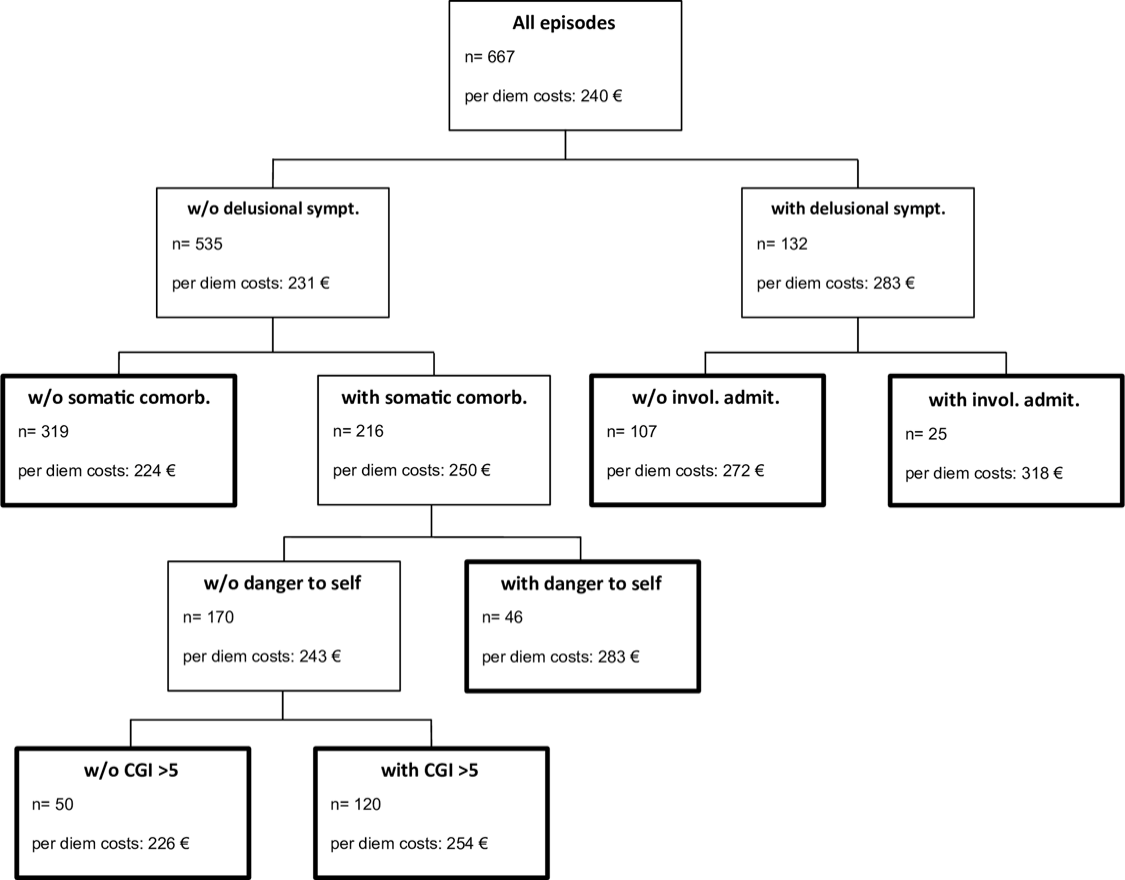
Conditional inference tree of per diem costs. W/O = Without, sympt. = symptoms, comorb. = comorbidities, invol. admit. = involuntarily admitted, CGI = Clinical Global Impression.

Fig 4 shows the single best conditional inference tree in the analysed data. The best cost split in the complete data set was found in the presence of delusional symptoms, followed by somatic comorbidities in patients without delusional symptoms and involuntary admission in patients with delusional symptoms. The tree-building procedure was configured to stop when the Bonferroni-adjusted tests of the global null hypothesis of no association between any of the covariates and the outcome could not be rejected at the 95% confidence level. The bold terminal nodes represent potential groups for payment purpose. The highest hospital costs were found in episodes with both delusional symptoms and involuntary admission. The lowest hospital costs were found in episodes with neither delusional symptoms, nor somatic comorbidities. The explained variance was 14% and the RMSE was 62.

## Discussion

The aim of this study was confirming the hypothesis that the eight concepts a priori identified as potential cost drivers in a systematic review [12] influence per diem hospital costs in a current European hospital setting. A second aim was exploring further patient variables that might influence hospital costs. The provided results confirmed the hypothesis that not being of middle age (33–64 years), danger to self, involuntary admission, problems in the activities of daily living, the presence of delusional symptoms, the presence of affective symptoms, short length of stay and the discharging ward affect per diem hospital costs. The explorative analysis found the GAF to be the most important variable in the prediction of per diem hospital costs in the analysed data, followed by delusional symptoms and referral from another hospital. A patient classification system for prospective per diem payment was suggested with the highest per diem hospital costs found in episodes having both delusional symptoms and involuntary admission and the lowest hospital costs in episodes having neither delusional symptoms nor somatic comorbidities. However, the explained variance was low (14%) and maybe insufficient for reimbursement purpose.

### Strength and weakness of the study

Strength of this study was the resource use data. These allowed a comprehensive and detailed calculation of mean per diem costs per episode. Furthermore, a work sampling study including all physicians, psychologists and nurses involved in clinical care during the study period allowed inclusion of an estimation of episode-specific per diem costs of regular care, which is usually difficult due to lack of data. A further strength was the reference to an a priori defined hypothesis, i.e. the influence of eight potential costs drivers, which should improve confidence in robustness of results.

A weakness of this study was its single site design. Although previous results were confirmed in this study, it still remains unclear whether inferences can be transferred to other clinical settings. For instance, the median length of stay in this study (51 days) was longer than the average length of stay in psychiatric hospitals in Germany and much longer than in some other health care systems, such as in the USA [22, 23]. In particular, the results of explorative nature, i.e. the variable importance (Fig 3) and the reimbursement groups (Fig 4), require tests of external validity in further, preferably multicentre studies.

A further weakness was in the daily documented acuity score used to calculate costs of regular care. This score has been developed by the German Institute of Hospital Reimbursement and the German Association for Psychiatry, Psychotherapy and Psychosomatics specifically to represent staff’s effort of regular care [24]. However, there have not been any studies of criterion validity carried out to date, which creates some uncertainty considering the valid representation of the effort of regular care. Nevertheless, the chosen two-stage approach of estimating costs of regular care, i.e. the initial measurement of total time per ward in the time study followed by allocation among patients according to the acuity score, should yield a more accurate estimate than available in most other studies.

### Results in relation to prior research

The hypothesis-driven analysis mainly confirmed the results of prior research [12], namely the effects of not being of middle age (33–64 years), danger to self, involuntary admissions, problems in the activities of daily living, the presence of psychotic symptoms or affective symptoms, short length of stay and treatment site. However, the main diagnoses showed neither substantial nor significant effects in the hypothesis-driven analysis. Furthermore, the main diagnoses were of low variable importance in the explorative analysis. This is in contrast to primary studies included in a recent systematic review, which found dementia and mood disorder patients consistently more costly than average and schizophrenia and substance-related disorder patients less costly than average [10,25,26]. An important difference in this study was that no patients with a primary diagnosis of dementia were included due to the acute care treatment focus. Moreover, the seven wards at the study site were non-exclusively dedicated to specific diagnostic groups and might have statistically absorbed the effects related to diagnoses. However, excluding the wards from the model did not increase the influence of main diagnoses on per diem costs (not shown in results).

Analyses in previous studies investigated the univariate influence of main diagnoses [10,25,26]. Removing the all other independent variables for a univariate analysis of main diagnoses and per diem costs in the study presented here yielded significant regression coefficients and a substantial share of explained variance of 6.5% (not shown in results). A potential explanation for differences in results between the previous studies and the study presented here is that the former did not control for other patient-related variables and length of stay, which might have been the real cost drivers.

The ensemble of conditional inference trees employed all 51 available patient variables. It explained the variance in per diem costs substantially to moderately in the complete sample (43.5%) and the internal cross-validation (19.4%), respectively. The absolute values of variable importance were neither informative themselves nor comparable to other studies. However, ten variables were identified that yielded substantially higher values than the other and these should constitute a basis for further investigations.

Interestingly, the most important variable in the ensemble of trees, the GAF score (Fig 3), was not statistically identified as relevant by the single conditional inference tree generated to create potential groups for payment purpose (Fig 4). In contrast to an ensemble of trees, where variables were randomly chosen at each split, single conditional inference trees compare all available variables at each split. Therefore, otherwise very important variables that were outplayed at a specific split might not enter the tree at all. The interested reader is recommended to find the paper of Strobl et al for a very accessible introduction to the applied methods [19].

The suggested patient classification system of six mutually exclusive and exhaustive groups for payment purpose can be employed with only five documented patient characteristics (Fig 4). Its model fit, as measured by the explained variance, was similar to the fit in previously developed classification systems of comparable parsimony [12]. However, in contrast to previous studies, it was based solely on patient variables and avoided tautological findings by excluding service variables that directly represent resource use. Moreover, overfitting was avoided by applying multiplicity-adjusted test procedures to find significant associations between any of the covariates and the response [17]. Ultimately, the explained variance might be too low and the RMSE might be too high to find acceptance as per diem payment system.

There are three potential reasons for relatively low power of patient variables in predicting mental health per diem resource use. First, there is a wider range of accepted treatment strategies and therefore less standardisation of care [4,27]. This could mean that resource allocation depends less on patient needs but more on different treatment philosophies. This would complicate the understanding of cost differences and would be coherent with the strong influence of treatment sites in previous studies [25,26,28] and wards in this study. Second, costs differences might have been more difficult to explain because they were in total smaller than in other medical disciplines. For instance, Buckingham et al [26] found that there was less variability between patient costs in the mental health system than in the broader health system of Australia. Therefore, they assumed that patient classifications in mental health, at best, could only be expected to explain a modest amount of cost variance. Third, existing cost drivers that could help to explain differences might have been unobserved and omitted from analysis. However, this appears unlikely with regard to the relatively large number of variables considered and the rather consistent results of low explanatory power among multiple studies.

### Implications and further research

Inappropriate hospital reimbursement can create unintended provider incentives and inefficient delivery of care [1–3]. The aim in prospective per diem payment is to achieve efficient delivery of care by setting adequate per diem rates. The results of this study supported the approach of using per diem rates that decline with increasing length of stay because they should follow the cost trajectories better than constant rates. However, although reliable cost drivers were identified, idiosyncrasies of mental health care complicated the identification of clear and consistent differences in per diem hospital costs between patient groups. Prospective per diem payment based on patient classification systems requires resource-homogeneous patient groups in order to set the right incentives required for the efficient delivery of inpatient mental health care. Further multicentre studies that might find algorithms to split patients in more resource-homogeneous groups are needed and would greatly inform current discussions about inpatient mental health reimbursement, for instance in the UK and Germany. If reimbursement does not follow hospital costs, classifications systems appear more useful for hospital statistics and benchmarking, but might not be reasonable for payment purpose.

## Supporting Information

S1 Fig Conditional variable importance of explored patient characteristics. AMDP = Association for Methodology and Documentation in Psychiatry, CGI = Clinical Global [29], Impression, km = kilometres, ADL = Activities of Daily Living, F-groups were derived from the, International Classification of Diseases German modification (ICD-10-GM).
(PDF)

S1 Table Additional independent variables used in explorative analysis. (PDF)
