# Supplementary material for: Determinants of per diem Hospital Costs in Mental Health
Source: PLoS One. 2016 Mar 31;11(3):e0152669. doi: 10.1371/journal.pone.0152669 (PMC4816317; doi:10.1371/journal.pone.0152669)
Supplement: S1 Fig — AMDP = Association for Methodology and Documentation in Psychiatry, CGI = Clinical Global [29], Impression, km = kilometres, ADL = Activities of Daily Living, F-groups were derived from the, International Classification of Diseases German modification (ICD-10-GM). (PDF) [file pone.0152669.s001.pdf]

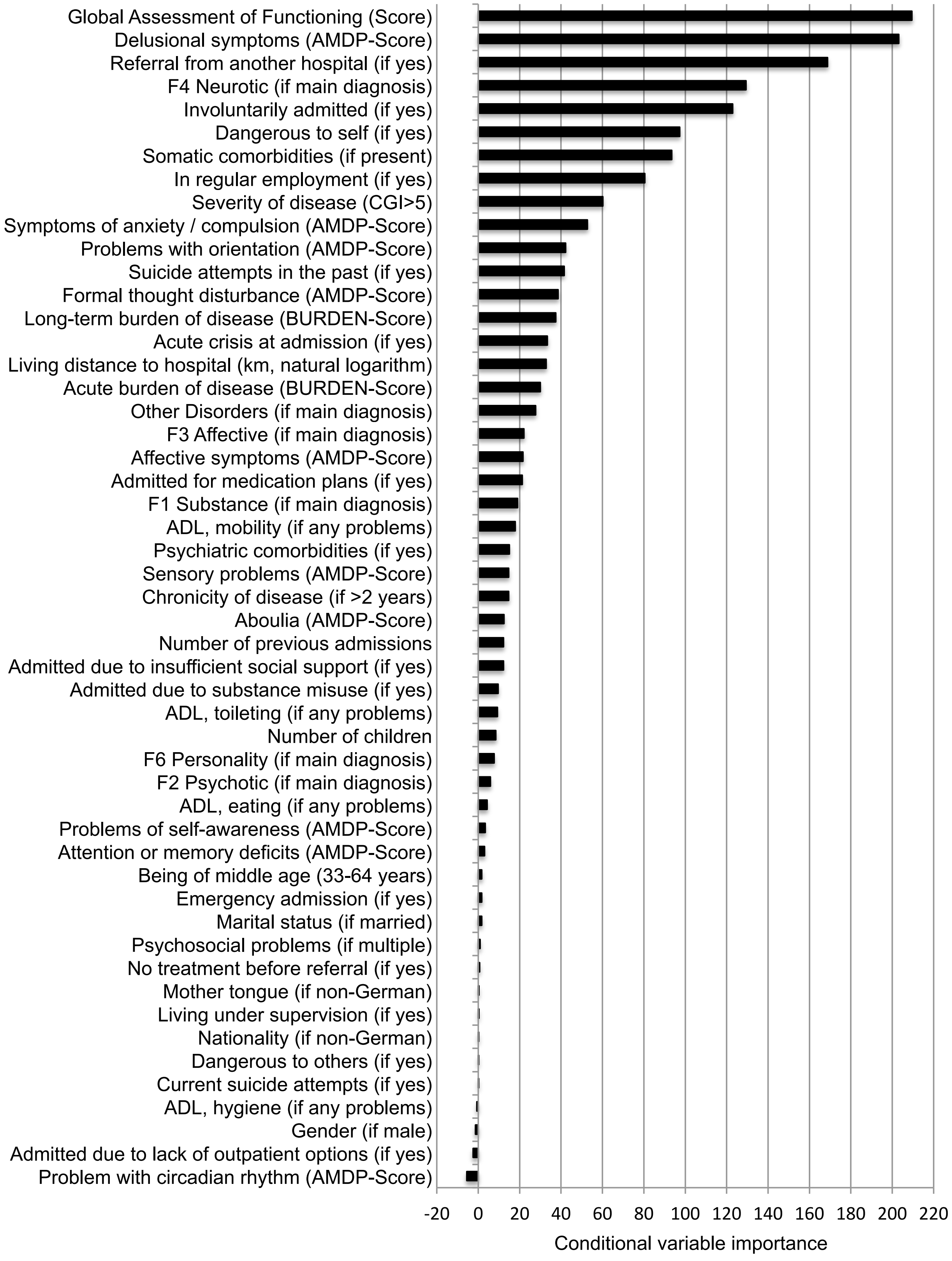

S2 Figure: Conditional variable importance of explored patient characteristics
AMDP= Association for Methodology and Documentation in Psychiatry, CGI= Clinical Global Impression, km= kilometres, ADL= Activities of Daily Living, F-groups were derived from the International Classification of Diseases German modification (ICD-10-GM)
