# Supplementary material for: Determinants of per diem Hospital Costs in Mental Health
Source: PLoS One. 2016 Mar 31;11(3):e0152669. doi: 10.1371/journal.pone.0152669 (PMC4816317; doi:10.1371/journal.pone.0152669)
Supplement: S1 Table — (PDF) [file pone.0152669.s002.pdf]

S1 Table: Additional independent variables used in explorative analysis

	n	%
Aboulia (AMDP-Score)		
not existent (0)	54	8
light (1)	142	21
medium (2)	223	33
severe (3)	242	36
missing	6	1
Acute burden of disease (BURDEN-Score)		
none (0)	127	19
light (1)	117	18
medium (2)	168	25
severe (3)	201	30
very severe (4)	35	5
missing	19	3
Acute crisis at admission		
no	380	57
yes	272	41
missing	15	2
Admitted due to insufficient social support		
no	490	74
yes	162	24
missing	15	2
Admitted due to lack of outpatient options		
no	112	17
yes	541	81
missing	14	2
Admitted due to substance misuse		
no	537	81
yes	116	17
missing	14	2
Admitted for medication plans		
no	278	42
yes	375	56
missing	14	2
Attention or memory deficits (AMDP-Score)		
not existent (0)	187	28
light (1)	235	35
medium (2)	190	29
severe (3)	49	7
missing	6	1
Chronicity of disease		
not more than 2 years	301	45
>2 years	361	54
missing	5	1
Current suicide attempts		
no	623	93
yes	30	5
missing	14	2

S1 Table: Additional independent variables used in explorative analysis (Continued)

	n	%
Emergency admission		
no	611	92
yes	42	6
missing	14	2
Formal thought disturbance (AMDP-Score)		
not existent (0)	272	41
light (1)	194	29
medium (2)	148	22
severe (3)	47	7
missing	6	1
Gender		
female	393	59
male	274	41
missing	0	0
Global Assessment of Functioning (Score)		
mean		42
median		43
min		7
max		90
standard deviation		12
IQR		17
missing		5
In regular employment		
no	450	68
yes	201	30
missing	16	2
Living distance to hospital (km)		
mean		57
median		19
min		0
max		699
standard deviation		99
IQR		49
missing		0
Living under supervision		
no	619	93
yes	34	5
missing	14	2
Long term burden of disease (BURDEN-Score)		
none (0)	55	8
light (1)	79	12
medium (2)	196	29
severe (3)	250	38
very severe (4)	68	10
missing	19	3
Marital status		
other	442	66
married	207	31
missing	18	3

S1 Table: Additional independent variables used in explorative analysis (Continued)

	n	%
Mother tongue		
German	593	89
non-German	60	9
missing	14	2
Nationality		
German	639	96
non-German	28	4
missing	0	0
No treatment before current admission		
no	602	90
yes	51	8
missing	14	2
Number of children		
0	366	55
1	81	12
2	133	20
3	44	7
4	19	3
5	10	2
missing	14	2
Number of previous admissions		
0	163	24
1	116	17
2	79	12
3	83	12
4	55	8
5	49	7
6	20	3
7	16	2
8	14	2
9	4	1
10	30	5
12	5	1
13	4	1
14	3	0
15	4	1
20	7	1
25	1	0
missing	14	2
Problems of self-awareness (AMDP-Score)		
not existent (0)	589	88
light (1)	72	11
medium (2)	0	0
severe (3)	0	0
missing	6	1
Problems with circadian rhythm (AMDP-Score)		
not existent (0)	333	50
light (1)	141	21
medium (2)	142	21
severe (3)	44	7
missing	7	1

S1 Table: Additional independent variables used in explorative analysis (Continued)

	n	%
Problems with orientation (AMDP-Score)		
not existent (0)	606	91
light (1)	55	8
medium (2)		
severe (3)		
missing	6	1
Psychiatric comorbidities		
no	331	50
yes	331	50
missing	5	1
Psychosocial problems		
one or less	433	65
multiple	229	34
missing	5	1
Referral from another hospital		
no	549	82
yes	104	16
missing	14	2
Sensory problems (AMDP-Score)		
not existent (0)	581	87
light (1)	27	4
medium (2)	34	5
severe (3)	19	3
missing	6	1
Severity of disease		
CGI<6	188	28
CGI>5	459	69
missing	20	3
Somatic comorbidities		
not present	387	58
present	275	41
missing	5	1
Suicide attempts in the past		
no	489	73
yes	164	25
missing	14	2
Symptoms of anxieties / compulsions (AMDP-Score)		
not existent (0)	233	35
light (1)	173	26
medium (2)	143	21
severe (3)	112	17
missing	6	1

AMDP= Association for Methodology and Documentation in Psychiatry, IQR= interquartile range, km= kilometres, CGI= Clinical Global Impression
